# Supramolecular architectures in multicomponent crystals of imidazole-based drugs and tri­thio­cyanuric acid

**DOI:** 10.1107/S2052520624005055

**Published:** 2024-07-01

**Authors:** Anna Ben, Marta Hoelm, Lilianna Chęcińska

**Affiliations:** a University of Lodz, Doctoral School of Exact and Natural Sciences, Narutowicza 68, 90-136Łódź, Poland; b University of Lodz, Faculty of Chemistry, Pomorska 163/165, 90-236Łódź, Poland; CSIR–National Chemical Laboratory, India

**Keywords:** crystal structure, synthon, supramolecular architecture, metronidazole, miconazole, ketoconazole, tri­thio­cyanuric acid, salt, cocrystal, cocrystal of salt

## Abstract

The structures of three multicomponent crystals formed with imidazole-based drugs, namely metronidazole, ketoconazole and miconazole, in conjunction with tri­thio­cyanuric acid are characterized. Each of the obtained adducts represents a different category of crystalline molecular forms: a cocrystal, a salt and a cocrystal of salt. The structural analysis revealed that in all cases, the N—H⋯N hydrogen bond is responsible for the formation of acid–base pairs, regardless of whether proton transfer occurs or not, and these molecular pairs are combined to form unique supramolecular motifs by centrosymmetric N—H⋯S interactions between acid molecules. The complex intermolecular forces acting in characteristic patterns are discussed from the geometric and energetic perspectives, involving Hirshfeld surface analysis, pairwise energy estimation, and natural bond orbital calculations.

## Abstract 2

The structures of three distinct forms (cocrystal, salt and a hybrid cocrystal of salt) of multicomponent crystals of imidazole-based drugs (metronidazole, ketoconazole and miconazole) with tri­thio­cyanuric acid as coformer are characterized. Their supramolecular architectures reflect the interplay between different molecular species.

## 1. Introduction

Crystal engineering, that is the design of desirable crystal architectures (Desiraju, 1989 ▸, 2007 ▸), has been attracting increasing attention for decades as it offers endless ways of creating new substances. Thanks to the combination of the wide range of ingredients and variety of synthetic methods, there has been rational development in this area (Haskins & Zaworotko, 2021 ▸). Recent years in particular have seen great interest in the use of cocrystallization methods to obtain multicomponent crystals (Haskins & Zaworotko, 2021 ▸; Saha *et al.*, 2023 ▸). Such complex systems, based on noncovalent interactions, are a source of fascination because of possibilities presented by combining several components with each other. Cocrystallization allows systems to be obtained consisting of two or more neutral molecules (cocrystals) or ionized species (salts) and their mixtures (including solvates or polymorphs) (Aitipamula *et al.*, 2012 ▸). All mentioned types of multicomponent crystalline structures containing an active pharmaceutical ingredient (API) have great potential in the pharmaceutical industry because (along with improved physicochemical properties) they essentially preserve or even enhance the inherent pharmacological properties of the active substance (Almarsson & Zaworotko, 2004 ▸; Yousef & Vangala, 2019 ▸; Bolla *et al.*, 2022 ▸). Among the APIs, some of the most frequently explored forms are combinations of those based on N-heterocyclic bases, such as imidazoles, with simple aromatic or aliphatic carb­oxy­lic acids (Martin *et al.*, 2013 ▸, 2020 ▸; Zheng *et al.*, 2019 ▸; Drozd *et al.*, 2021 ▸). Imidazole-based compounds are of particular interest in medicinal chemistry as they can have properties such as anticancer, antifungal, antibacterial, antitubercular, anti-inflammatory, antineuropathic, antihypertensive, antihistaminic, antiparasitic, antiobesity, antiviral, and others (Zhang *et al.*, 2014 ▸; Tolomeu & Fraga, 2023 ▸). This study explores the multicomponent crystals of three imidazole-containing drugs, namely, metronidazole (MTZ), ketoconazole (KTZ) and miconazole (MIC), with an effective cocrystallizing agent, tri­thio­cyanuric acid (TTCA) (see Scheme 1).
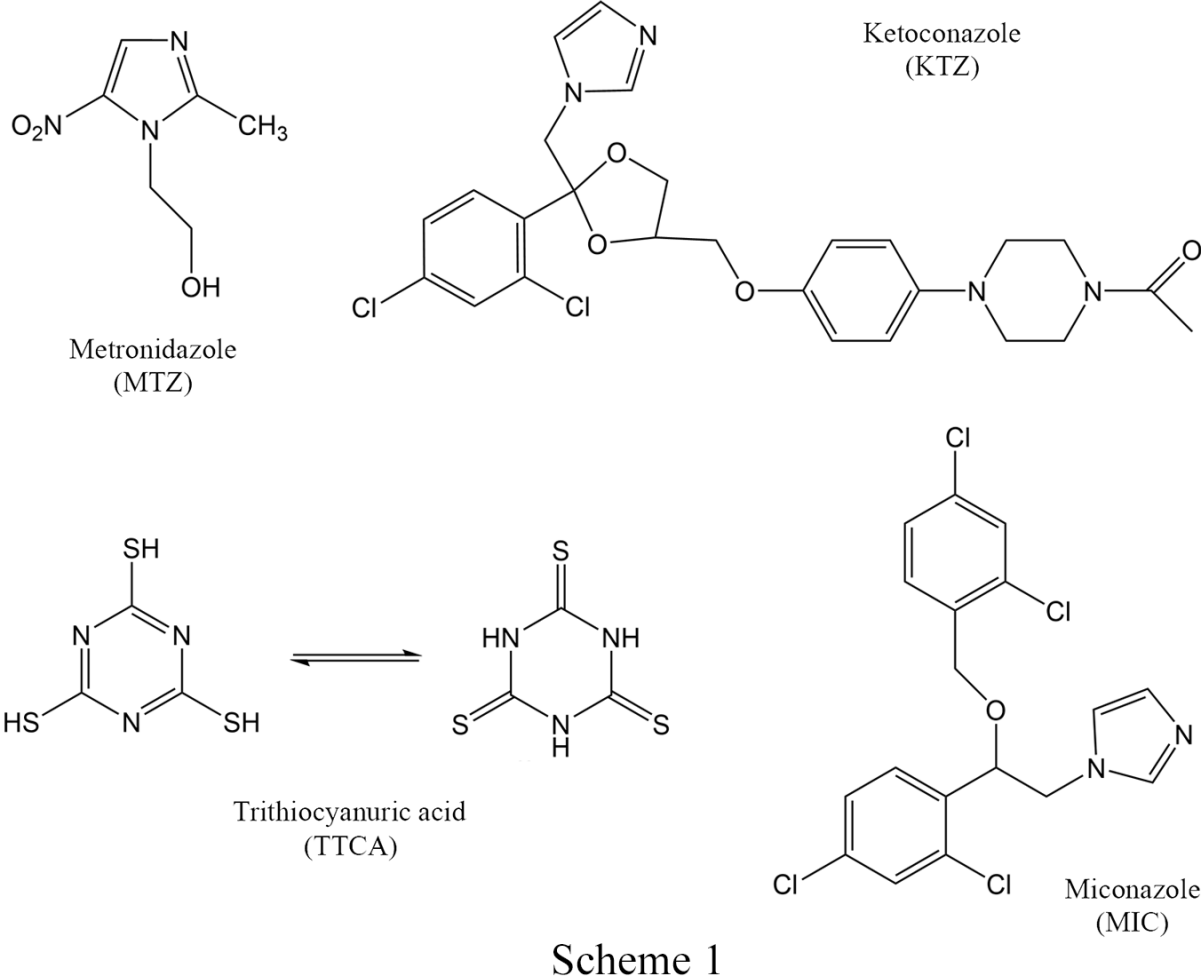


Metronidazole {MTZ; C_6_H_9_N_3_O_3_; CAS No. 443-48-1; systematic name: 1-(2-hy­droxy­ethyl)-2-methyl-5-nitro­imidazole} is a water-soluble drug with antiprotozoal and antibacterial activities (Li *et al.*, 2021 ▸). MTZ is on the World Health Organization Model List of Essential Medicines (https://www.who.int/publications/i/item/WHO-MHP-HPS-EML-2023.02), and enjoys widespread use, especially as a first-line therapy for anaerobic infections and for infections caused by *Trichomonas* (Sobel & Sobel, 2015 ▸).

Ketoconazole {KTZ; C_26_H_28_Cl_2_N_4_O_4_; CAS No. 65277-42-1; systematic name: *cis*-1-acetyl-4-[4-[[2-(2,4-di­chloro­phenyl)-2-(1*H*-imidazole-1-ylmethyl)-1,3-dioxolan-4-yl]meth­oxy]-phenyl]piperazine} is an antifungal drug with a broad spectrum of activity, used in the treatment of a wide range of superficial mycoses (Odds *et al.*, 1980 ▸; Delgado *et al.*, 1998 ▸).

Miconazole {MIC; C_18_H_14_Cl_4_N_2_O; CAS No. 22916-47-8; systematic name: (*RS*)-1-[2-(2,4-di­chloro­benzyl­oxy)-2-(2,4-di­chloro­phenyl)­ethyl]-1*H*-imidazole}. It is currently one of the most widely used antifungal drugs. MIC is commonly used to treat mucosal dermatophytes, yeast and Gram-positive bacteria infections (Botter, 1971 ▸; Sawyer *et al.*, 1975 ▸; Nenoff *et al.*, 2017 ▸).

Tri­thio­cyanuric acid {TTCA; C_3_H_3_N_3_S_3_; CAS No. 638-16-4; systematic name: 1,3,5-triazine-2,4,6(1*H*,3*H*,5*H*)-tri­thione}, also called trimercaptotriazine, is a compound with a symmetrical structure, which has hydrogen-bond donor and hydrogen-bond acceptor groups. It is therefore mostly used for the construction of coordination networks, for which the antitumor, antimicrobial and antibacterial activities have been evaluated (Kopel *et al.*, 2007 ▸, 2014 ▸, 2015 ▸).

The unique structural motifs formed between imidazole-containing drugs and TTCA coformer molecules are promising for the crystal engineering of similar systems; our study therefore looks more closely at the crystal architecture of new multicomponent modifications. It thus provides new insights into the rational design of drugs using potential *N*-donor and *N*-acceptor groups. Our observations are supported by analyses of structural motifs from the geometrical and energetic point of view, and the structural findings are supported by an natural bond orbital (NBO) analysis of the optimized gas-phase structures of TTCA–coformer molecules.

## 2. Experimental

### 2.1. Synthesis and crystallization

The following substances were used in this study: metronidazole (purity > 99%, TCI: Tokyo Chemical Industry, Japan), ketoconazole (purity > 98%, TCI, Japan), miconazole (purity > 99%, Fluoro­chem, UK) and tri­thio­cyanuric acid (purity > 98%, TCI, Japan).

Preparation of MTZ·TTCA. Equimolar amounts (0.05 mmol in a 1:1 molar ratio) of metronidazole and tri­thio­cyanuric acid were dissolved in a water–methanol (1:2, *v*/*v*) solution, and left to evaporate in the refrigerator. Crystals suitable for X-ray measurements were obtained in two weeks.

Preparation of KTZ(+)·TTCA(−)·0.16H_2_O. Ketoconazole (0.004 g) and tri­thio­cyanuric acid (0.001 g) were mixed in ethanol (3 ml) and left to evaporate slowly at room temperature. After one week, crystals suitable for X-ray measurements were obtained.

Preparation of MIC·MIC(+)·TTCA(−). Equimolar amounts (0.05 mmol in a 1:1 molar ratio) of miconazole and tri­thio­cyanuric acid were mixed in ethanol (3 ml) and left to evaporate slowly at room temperature. After two weeks, crystals suitable for X-ray measurements were obtained.

### 2.2. Refinement

Crystal data, data collection and structure refinement details for the three compounds are summarized in Table 1 ▸. The H atoms bonded to C atoms were included in the refinement using riding models, with constrained distances set to 0.95 Å (aromatic), 0.98 Å (*R*–CH_3_), 0.99 Å (*R*_2_–CH_2_) and 1.0 Å (*R*_3_–CH) (for *R* = C,N,O), and with *U*_iso_(H) = 1.2*U*_eq_ or 1.5*U*_eq_ (for *R*–CH_3_ only) of the attached C atom.

The hydrogen atoms bonded to heteroatoms (N, O), involved in hydrogen bonds, were located in difference Fourier maps and refined freely.

During the refinement of KTZ(+)·TTCA(−)·0.16H_2_O, the (1,3-dioxolan-4-yl)meth­oxy fragment was found to be disordered and refined with two alternative positions with the final site-occupancy factors *A* to *B* components: *k*_*A*_:*k*_*B*_ = 0.625 (7):0.375 (7). Additionally, geometrical similarity constraints were applied to the O3*A*—C15 and O3*B*—C15 bond lengths using the SADI instruction (in *SHELXL*). The RIGU command (in *SHELXL*) was used to maintain the structural integrity of disordered groups.

In KTZ(+)·TTCA(−)·0.16H_2_O, one partially occupied water molecule is present in the crystal structure. The occupancy ratio was refined to 0.160 (8); (O)−H atoms were constrained (AFIX 6) and their *U*_iso_ was fixed to 1.5*U*_eq_(O). The stoichiometry of water was identified by occupancy refinement of electron density located in a solvent-accessible pocket in close proximity to the disordered (1,3-dioxolan-4-yl)­meth­oxy fragment. This partially occupied water is engaged in the hydrogen-bonding network.

### 2.3. Hirshfeld surface analysis

Hirshfeld surfaces and fingerprint plots (Spackman & McKinnon, 2002 ▸; Spackman & Jayatilaka, 2009 ▸) were generated using *CrystalExplorer* software (Spackman *et al.*, 2021 ▸).

### 2.4. Pairwise model energies

*CrystalExplorer* software was used to estimate pairwise model energies (Turner *et al.*, 2014 ▸) between molecules within clusters: within a radius of 5 Å, for a cocrystal of MTZ–TTCA or 25 Å, for a salt of KTZ(+)·TTCA(−)·0.16H_2_O and a cocrystal of salt of MIC·MIC(+)·TTCA(−) (Spackman *et al.*, 2021 ▸). The computational approach uses a B3LYP/6-31G(d,p) molecular wavefunction calculated for the respective molecular arrangement in the crystal. The total interaction energy between any nearest-neighbour molecular pairs was estimated in terms of four components: electrostatic, polarization, dispersion and exchange-repulsion; the calculation employed scale factors of 1.057, 0.740, 0.871 and 0.618, respectively.

Please note that the energy values computed for ionized pairs should be interpreted with care, and thus the approximate set of energies is used to show the energetic trends rather than exact values.

### 2.5. Theoretical calculations

Full geometry optimizations of the tri­thio­cyanuric acid molecule [TTCA] and an anion [TTCA(−)], and of the corresponding hydrogen-bonded dimers TTCA–TTCA, TTCA(−)–TTCA(−)_*ortho* and TTCA(−)–TTCA(−)_*para* were performed using the density functional theory (DFT) method with *Gaussian16* software (revision C.02; Frisch *et al.*, 2016 ▸). The calculations were conducted in the gas phase at the M06L/6-311++G(3df,3pd) level of theory; the meta exchange-correlation functional M06L is known to have comparable accuracy to CCSD calculations performed on small non-covalently interacting systems (Remya & Suresh, 2013 ▸). The harmonic vibrational calculations indicate an absence of imaginary frequencies, thus confirming that the analysed molecules and dimers are true minima on the potential energy surface.

Natural atomic charges and intermolecular donor–acceptor orbital interactions were determined based on NBO calculations, which were performed at the same level of theory as the geometry optimizations. The significance of the orbital interactions was quantified by the stabilization energy *E*(2) associated with the electron delocalization from the donor orbital (*i*) to the acceptor (*j*). This energy was assessed using second-order perturbation theory, as follows:
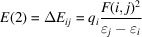
where *q_i_* is the donor orbital occupancy, ɛ_*j*_,ɛ_*i*_ is the diagonal elements (orbital energies), *F*(*i*,*i*) is the off-diagonal NBO Fock matrix element (Foster & Weinhold, 1980 ▸; Reed & Weinhold, 1983 ▸; Reed *et al.*, 1985 ▸). The NBO calculations were performed using *NBO3.1* software implemented in the *Gaussian16* package.

## 3. Results and discussion

### 3.1. Cocrystal and salt, and their hybrid

Among multicomponent crystals, cocrystals and salts can be distinguished by the location of the proton between an acid and a base in their structure. A salt is formed by proton transfer from an acid to a base; in this case, ionized molecules are present in the crystal structure; while for a cocrystal, no proton transfer is observed, the proton remains on the acid, and the crystal is composed of neutral molecules. In this study, the location of the hydrogen atom was confirmed from the difference Fourier maps of the single-crystal structures. Thus, both neutral metronidazole (MTZ) and tri­thio­cyanuric acid (TTCA) molecules were found to form a cocrystal, whereas the ketoconazole [KTZ(+)] and tri­thio­cyanuric acid [TTCA(−)] molecules formed a salt; these were ionized by the transfer of one proton from TTCA to the basic imidazole-N atom of KTZ. Additionally, the presence of a partially occupied water molecule, 0.16 (1)H_2_O, indicates the salt is hydrated.

The multicomponent crystal of the third drug, miconazole, is not a typical cocrystal or salt by definition: its asymmetric unit consists of ionized species of miconazole [MIC(+)] and tri­thio­cyanuric acid [TTCA(−)] through analogous proton transfer to that observed in the KTZ salt. However, the crystal structure also contains an additional neutral miconazole molecule (MIC); such a ternary adduct is difficult to categorize. While the debate regarding such classification remains on-going, such molecular complexes can for now be classified as a cocrystal of a salt or a cocrystal salt (Aitipamula *et al.*, 2012 ▸; Odiase *et al.*, 2015 ▸; Grothe *et al.*, 2016 ▸; Zhoujin *et al.*, 2022 ▸), or a salt cocrystal (or a salted cocrystal) (Cherukuvada *et al.*, 2013 ▸; Yu *et al.*, 2021 ▸; Zhang *et al.*, 2023 ▸). In this study, the multicomponent crystal of MIC·MIC(+)·TTCA(−) is classified as a cocrystal of a salt.

There is also another terminology, ionic cocrystals (ICs), which can be used for mixed species observed in MIC·MIC(+)·TTCA(−). Although originally ionic cocrystals were formed by ionic salts (with an inorganic counterion) and an organic molecule (Braga *et al.*, 2010 ▸, 2011 ▸), recently this concept has been applied to organic salts and a neutral conformer (Wang *et al.*, 2018 ▸; Rahmani *et al.*, 2022 ▸). However, this nomenclature seems to be less used in the literature.

Additionally, in all studied drug molecules, the imidazole N atom was confirmed to be protonated, as indicated by the analysis of the C—N—C angle in the heterocyclic ring. The C—N—C angle in the neutral metronidazole molecule was found to be 105.74 (11)°, which is similar to that found in the neutral miconazole moiety, 105.0 (2)°; however, both values differ significantly from those found in ionized molecules of miconazole and ketoconazole, these being 109.4 (2)° and 107.9 (3)°, respectively. The obtained C—N—C angle values correspond well with the geometry of the imidazole ring found in the various crystal structures of MTZ, MIC and KTZ deposited in the Cambridge Structural Database (CSD version 5.44, September 2023; Groom *et al.*, 2016 ▸). For the analysed imidazole-based drugs, the cationic forms were found to exhibit higher values (109–111°) than the corresponding neutral moieties (103–106°) (Figs. S1–S3 in the supporting information). Only one exception from the rule has been recognized: the first determination of the miconazole hemihydrate from 1979 (MICONZ; Peeters *et al.*, 1979 ▸) indicated extremely high C—N—C angles in two independent neutral molecules, these being 107.260° and 108.516°.

In addition, while the formation of cocrystals and salts can be predicted by the Δp*K*_a_ rule, [Δp*K*_a_ = p*K*_a_(base) − p*K*_a_(acid)], this approach cannot be used for testing the multicomponent crystals considered in this study. According to this empirical rule, the cocrystal formation can be expected at Δp*K*_a_ < −1 and salt formation at Δp*K*_a_ > 4; however, acid–base pairs with Δp*K*_a_ values that lie in-between are difficult to clearly classify (Cruz-Cabeza, 2012 ▸). As such, based on the calculated Δp*K*_a_ values for the MTZ (p*K*_a_ = 2.62), KTZ (p*K*_a_ = 6.51) and MIC (p*K*_a_ = 6.91) imidazole bases and tri­thio­cyanuric acid (p*K*_a_ = 6.35), only the TTCA–MTZ pair meets the criterion for cocrystal formation (Δp*K*_a_ = −3.73). The remaining two acid-base pairs, namely, TTCA–KTZ and TTCA–MIC, have Δp*K*_a_ values close to 0. Interestingly, the literature p*K*_a_ value for MTZ differs considerably from those of KTZ and MIC; this reflects a fundamental difference between the substituted nitro­imidazole base moiety of MTZ and the pure (unsubstituted) imidazole base of KTZ and MIC drugs.

### 3.2. Molecular structures of imidazole-based drugs

**Figure 1:**
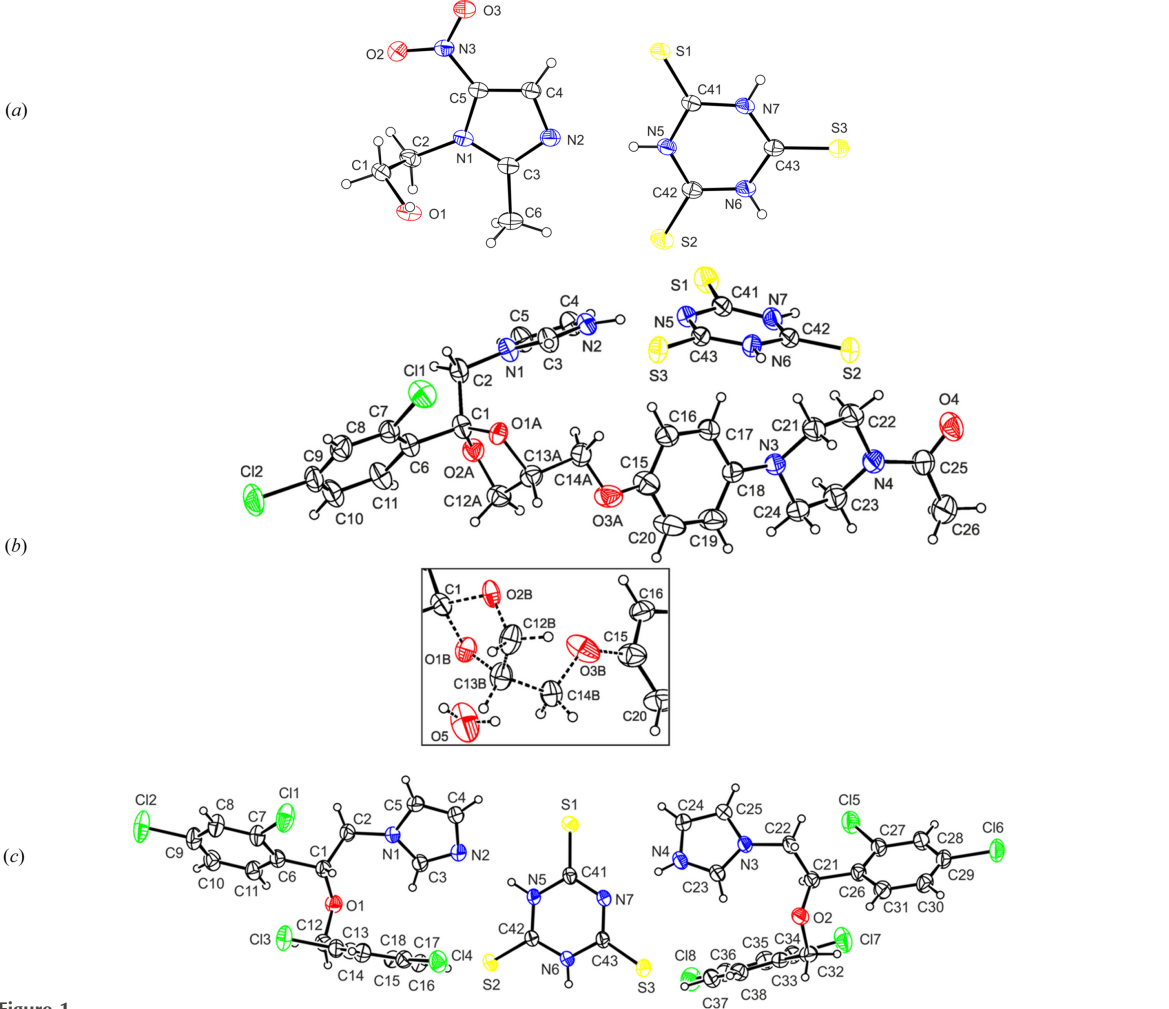
Views of the asymmetric unit of (*a*) MTZ·TTCA, (*b*) KTZ(+)·TTCA(−)·0.16H_2_O and (*c*) MIC·MIC(+)·TTCA(−), with the atom-numbering schemes. In KTZ(+)·TTCA(−)·0.16H_2_O, the major disorder component is drawn using unbroken lines (atoms with *A* suffix) and the minor disorder component is drawn using dashed lines (atoms with *B* suffix). Although the partially occupied water molecule is only shown with the minor component representation, it is associated with both orientations of the disordered group. Displacement ellipsoids are drawn at the 30% probability level. H atoms are shown as spheres of arbitrary radii.

The asymmetric units of the three considered structures of imidazole-based drugs with tri­thio­cyanuric acid, namely, MTZ·TTCA, KTZ(+)·TTCA(−)·0.16H_2_O and MIC·MIC(+)·TTCA(−), are given in Figs. 1 ▸(*a*)–1 ▸(*c*). In the MTZ·TTCA cocrystal, the geometry of the metronidazole molecule is in agreement with that previously reported by Kalaiarai *et al.* (2019 ▸) and is not discussed here.

In KTZ(+)·TTCA(−)·0.16H_2_O salt, the ketoconazole cation exists as a racemic mixture of two enantiomers, (2*S*,4*R*)-(−)-ketoconazole and (2*R*, 4*S*)-(+)-ketoconazole, represented by *A* and *B* components of the disorder of the (1,3-dioxolan-4-yl)meth­oxy fragment. For this, the final site occupancy factors were refined as *k*_*A*_:*k*_*B*_ = 0.625 (7):0.375 (7). The KTZ molecule has two stereocenters on the C1 and C13*A*/*B* atoms. The disordered 1,3-dioxolane ring is twisted on O2*A*—C12*A*/C1—O2*B* for the *A*/*B* components, with asymmetry parameters (Duax & Norton, 1975 ▸) of ΔC_2_(O2*A*—C12*A*) = 4 (2)° and ΔC_2_(C1—O2*B*) = 3 (2)°, respectively. The piperazine ring adopts a chair conformation with puckering parameters (Cremer & Pople, 1975 ▸) of *Q* = 0.525 (4) Å, θ = 180.0 (4)°, φ = 158 (15)°.

In the cocrystal of salt, MIC·MIC(+)·TTCA(−), a root-mean-square (RMS) deviation of 0.183 Å (Spek, 2020 ▸) was calculated for an overlay of 25 non-hydrogen atoms of two MIC molecules (one neutral and one ionized in the asymmetric unit. This reflects a high similarity between the two independent miconazole molecules (one neutral and one ionized) in the asymmetric unit. A molecular overlay is presented in Fig. S4.

### 3.3. Supramolecular motifs

**Figure 2:**
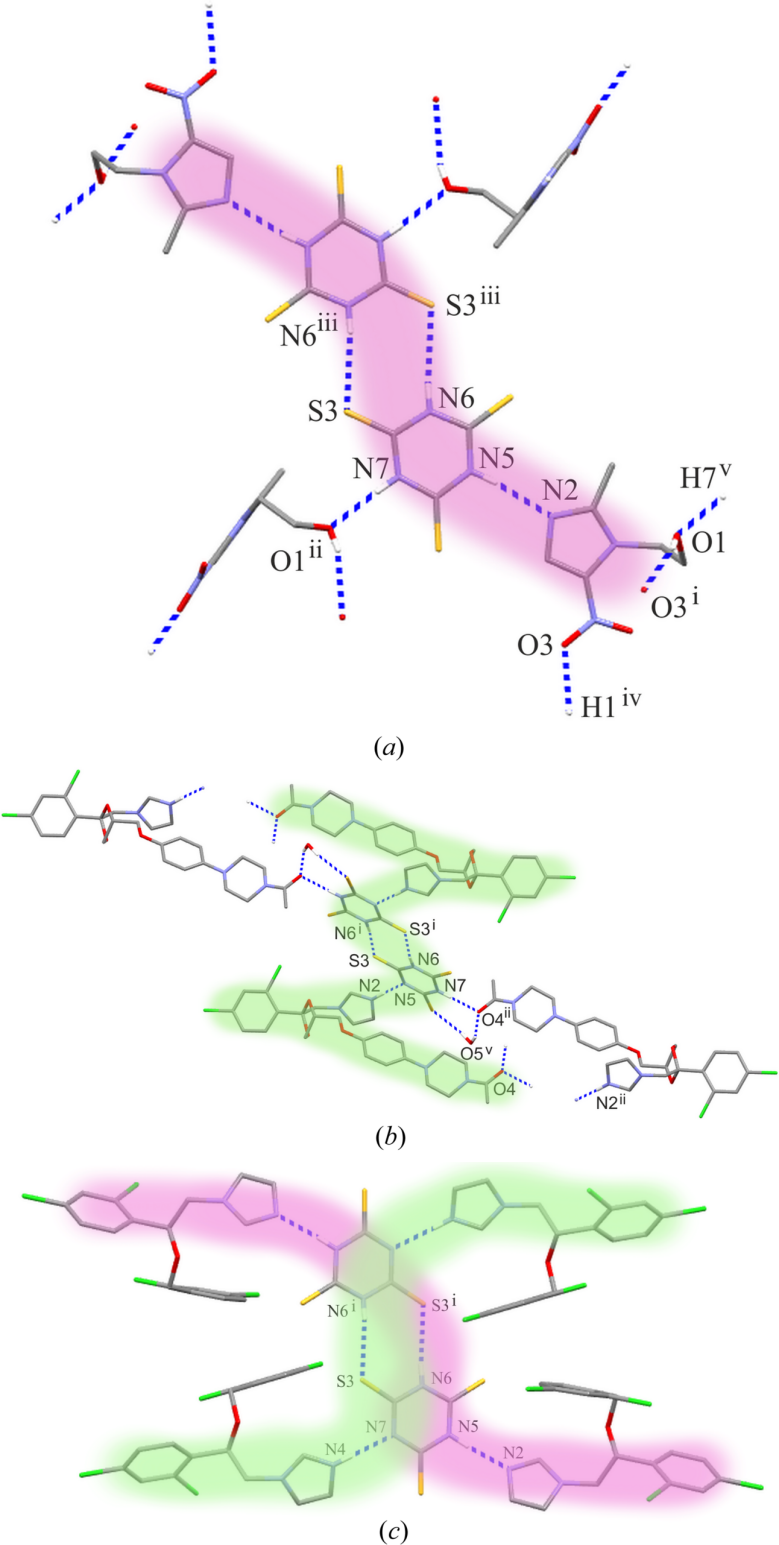
Parts of the crystal structure of (*a*) MTZ·TTCA, (*b*) KTZ(+)·TTCA(−)·0.16H_2_O and (*c*) MIC·MIC(+)·TTCA(−) showing supramolecular motifs marked in colour. Symmetry codes: MTZ·TTCA (i) −*x* + 1, *y* − ½, −*z* + ½; (ii) *x*, −*y* − ½, *z* − ½; (iii) −*x*, −*y* − 2, −*z*; (iv) −*x* + 1, *y* + ½, −*z* + ½; (v) *x*, −*y* − ½, *z* + ½; KTZ(+)·TTCA(−)·0.16H_2_O (i) −*x* + 1, −*y*, −*z*; (ii) −*x* + 2, −*y* + 1, −*z*; (v) −*x* + 1, −*y* + 1, −*z*; MIC·MIC(+)·TTCA(−) (i) − *x* + 1, −*y*, −*z* + 1.

**Figure 3:**
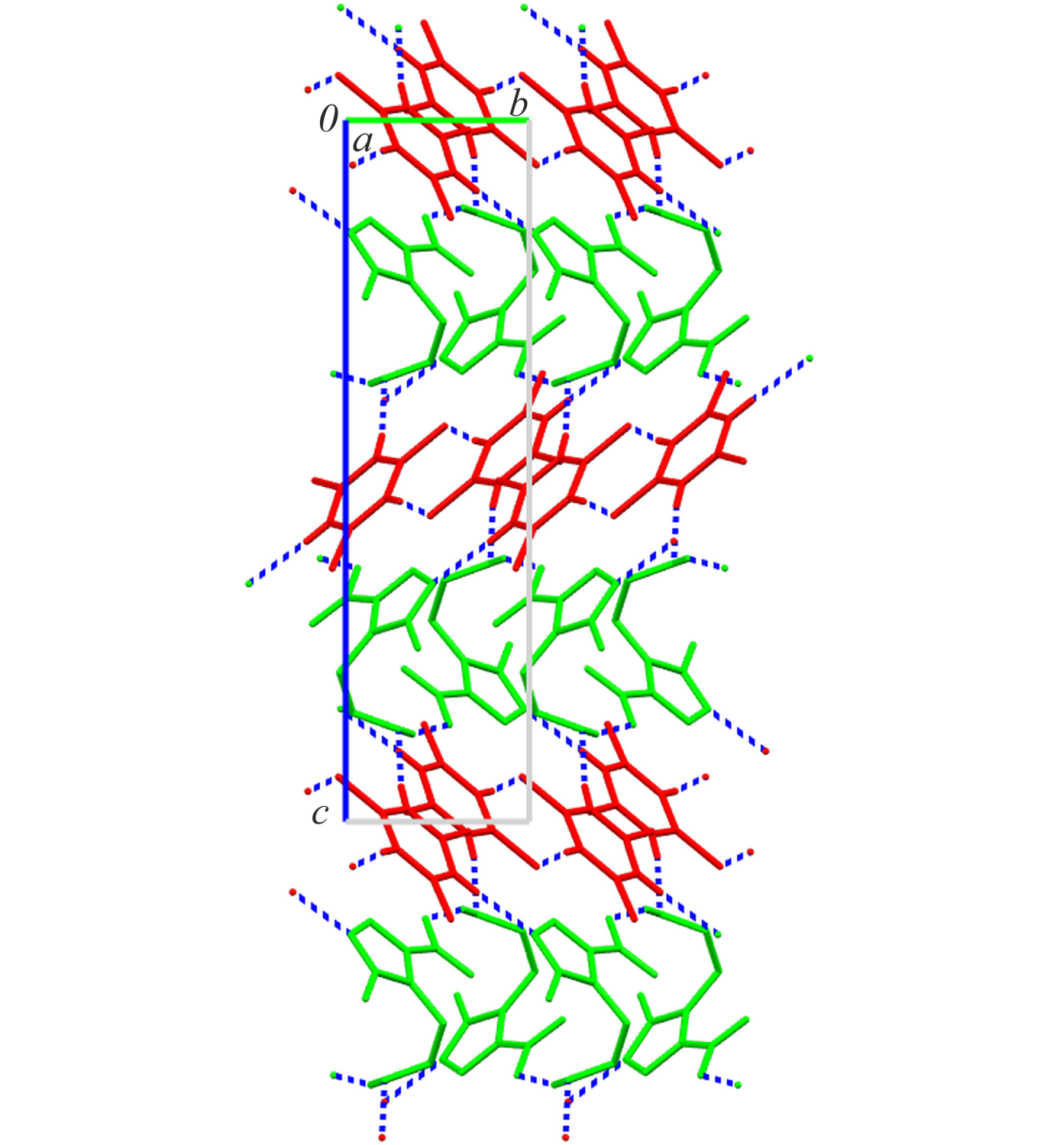
Crystal packing of MTZ·TTCA cocrystal in a view along the crystallographic *a* axis showing a layer-cake structure; the colour code is green = MTZ and red = TTCA.

In the cocrystal of MTZ·TTCA, the neutral molecules of the asymmetric unit interact with each other through the almost linear N5—H5⋯N2 hydrogen bond (Table 2 ▸). Tri­thio­cyanuric acid acts as a donor, while the basic imidazole-N atom of metronidazole acts as an acceptor. The centrosymmetric interaction of N6—H6⋯S3(−*x*, −*y* − 2, −*z*) between TTCA molecules combines two acid-base pairs into a unique supramolecular motif, shown in pink in Fig. 2 ▸(*a*). In the crystal structure, this four-molecule motif is further propagated by two intermolecular interactions, N7—H7⋯O1(*x*, −*y* − ½, *z* − ½) and O1—H1⋯O3(−*x* + 1, *y* − ½, −*z* + ½); therefore, the final supramolecular architecture of MTZ·TTCA is tri-periodic. The drug and conformer molecules form an alternating packing structure which resembles a layer-cake structure (Fig. 3 ▸). A weak C4—H4⋯S1 contact (Table 2 ▸), not indicated in any figure, supports the asymmetric acid–base pair.

In the KTZ(+)·TTCA(−)·0.16H_2_O salt, the cation and anion from the asymmetric unit are linked by a charge-assisted N2(+)—H2⋯N5(−) hydrogen bond, for which the protonated imidazole-N atom of KTZ acts as a donor (Table 3 ▸). Two ionic pairs are joined into a supramolecular motif [presented in green in Fig. 2 ▸(*b*)] by the centrosymmetric N6—H6⋯S3(−*x* + 1, −*y*, −*z*) interaction between two TTCA anions. The C—H⋯O contacts propagate the characteristic motif into a tri-periodic network. A partially occupied water molecule can also be seen in the crystal structure: it appears approximately three times every 20 unit cells. This water molecule does not disturb the formation of the supramolecular motif, being incorporated into the gaps between the molecules interacting with TTCA anions and KTZ cations *via* O5—H5*A*⋯O4(−*x* + 1, −*y* + 1, −*z*) and O5—H5*B*⋯S1(*x*−1, *y*, *z*) hydrogen bonds. If water were not included in the crystal structure, the supramolecular motifs would cluster into (001) di-periodic layers.

**Figure 4:**
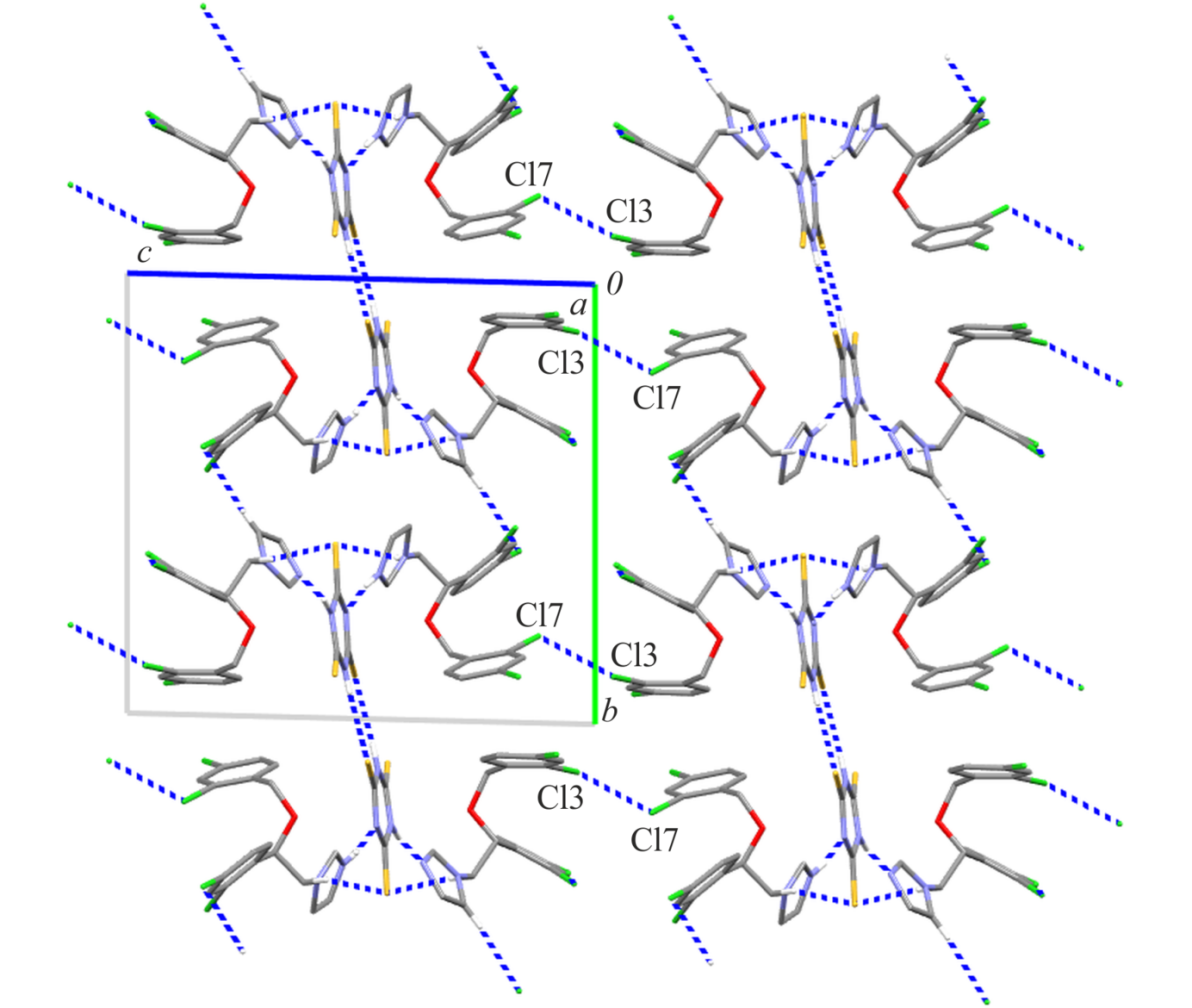
The crystal packing of MIC·MIC(+)·TTCA(−) showing the C14—Cl3⋯Cl7 halogen bond between di-periodic sheets; a view along the crystallographic *a* axis.

In the MIC·MIC(+)·TTCA(−) cocrystal of salt, the supramolecular motifs found in the MTZ·TTCA cocrystal and KTZ(+)·TTCA(−)·0.16H_2_O salt are combined with each other. On one side, the TTCA anion interacts with the neutral MIC molecule *via* the N5—H5*A*⋯N2 interaction, and from the other, with the MIC cation by the charge-assisted N4(+)—H4*A*⋯N7(−) hydrogen bond, acting as a donor and acceptor (Table 4 ▸). Similarly, the centrosymmetric N6—H6*A*⋯S3(−*x* + 1, −*y*, −*z* + 1) interaction between the TTCA anions is preserved. The coexistence of two single motifs in one common finite pattern is illustrated in Fig. 2 ▸(*c*). Three C—H⋯S/Cl contacts link the adjacent clusters to (001) di-periodic sheets. The C14—Cl3⋯Cl7(*x* + 2, *y*, *z* − 1) halogen bond extends the supramolecular structure to a tri-periodic assembly (Fig. 4 ▸); the distance of the close Cl3⋯Cl7 contact is 3.432 (2) Å, and the C14—Cl3⋯Cl7 angle is 156.9 (1)°.

On the other hand, in the case of MIC·MIC(+)·TTCA(−) cocrystal of salt (or ionic cocrystal), the presence of a neutral MIC molecule can be justified by donor–acceptor mismatch compared to the MTZ cocrystal and the KTZ salt. The N atom in the imidazole ring of the neutral miconazole molecule plays an important role as the only strong acceptor of the N—H⋯N hydrogen bond, replacing the oxygen atom in the N—H⋯O interactions in MTZ and KTZ crystals reported here. Summarizing, an additional molecule of MIC is required in the crystal structure to complement and stabilize the supramolecular motif built by the ionic pair.

### 3.4. Hirshfeld surface analysis

To provide further insight into the packing and intermolecular contacts in the analysed structures of multicomponent crystals, the imidazole-based drugs and TTCA-coformer molecules were separately subjected to Hirshfeld surface analysis. Figs. S5 and S6 show the respective percentage contributions of various intermolecular contacts to the Hirshfeld surface area for the two types of molecules. At first glance, it can be seen that the metronidazole moiety has a Hirshfeld fingerprint breakdown different from that of the ketoconazole and miconazole molecules; this is due to the fact that a variety of functional groups (nitro, hy­droxy­ethyl and methyl) are substituted into the imidazole ring of MTZ in contrast to an unsubstituted imidazole moiety and a few other ring fragments in KTZ and MIC drugs. Therefore, the O⋯H contacts represent one of the most important groups on the Hirshfeld surface of MTZ; they are less important in KTZ and disappear for MICs. In turn, contacts with terminal chlorine atoms, such as Cl⋯H, Cl⋯Cl or C⋯Cl, play a dominant role on the Hirshfeld surface of both miconazole molecules, constituting 49.7% of the surface for MIC and 40.7% for MIC(−), and are also noticeable for KTZ (∼16%). In all cases, the next most dominant group of close interactions (30–50%) is represented by H⋯H and C⋯H contacts, although in variable proportions. Surprisingly, S⋯H contacts make a significant contribution to the MTZ area; however, this may be due to the relatively smaller number of atoms and, therefore, the smaller surface area of the MTZ molecule compared to other drugs.

The breakdown diagram of contact shares for TTCA differs between the examined structures: the S⋯H contacts clearly dominate, the percentage of H⋯H maintains stable, but the remaining shares (∼40%) are significantly diversified.

An analysis of normalized contact distances in two-dimensional fingerprint plots (Figs. S7 and S8) indicates that O⋯H and N⋯H interactions compete with each other to be the shortest for the drug molecules, whereas the S⋯H contacts also become important for TTCA molecules.

In summary, it is difficult to identify trends resulting from the similarity of supramolecular motifs. Rather, differences are observed because of different tri-periodic crystal architectures.

### 3.5. Pairwise model energies

These findings raise the question of the role of hydrogen-bonded motifs in the formation of the supramolecular architecture of multicomponent crystals from the viewpoint of the energy of the intermolecular interactions.

The interaction energies for the most important molecular pairs in the examined crystal structures are given in Table 5 ▸. In a cocrystal form of MTZ·TTCA, the molecular pair connected by the N—H⋯O(imidazole) hydrogen bond has slightly higher energy than the pair from the asymmetric unit (TTCA–MTZ) binding through the N—H⋯N(imidazole) hydrogen bond, which has a less negative value; in third position is the MTZ–MTZ pair with the O—H⋯O hydrogen bond. However, the difference between the first and last positions does not exceed 5 kJ mol^−1^. It can clearly be seen that the magnitudes of the total energies differ significantly between completely ionized species in a salt and the cocrystal form. The highest attractive pairwise energy is nearly eight times greater for the acid–base pair connected by the N(+)−H⋯N(−) charge-assisted hydrogen bond (∼−400 kJ mol^−1^) than for the neutral form. The hybrid structure of the cocrystal of salt, MIC·MIC(+)·TTCA(−), is confirmed by the observed energetic trends, *i.e.* −407 kJ mol^−1^ for pairs of molecules with proton transfer compared to −43 kJ mol^−1^ for those without. Thus, it seems that the energetic hierarchy in salts and salt adducts is more varied, and appears to be more transparent than in cocrystals.

In the case of TTCA molecules which are centrosymmetrically related through the N—H⋯S hydrogen bond, those built from neutral molecules have a much lower total attractive energy (−26.6 kJ mol^−1^) compared to the acid–base pair in the same cocrystal form, while dimers of TTCA(−) anions are characterized by a repulsive energy around 150 kJ mol^−1^.

### 3.6. NBO calculations for the TTCA coformer

The study also examined the preferred site of deprotonated nitro­gen atom in TTCA, *i.e.* a site where the proton transfer occurs, in respect to the hydrogen-bonded synthon in the dimer formed by TTCA anions. This was confirmed using NBO analysis, which allows atomic charges to be calculated. As the charge acquired by an atom reflects the extent of electron repositioning, an analysis of atomic charges can provide an insight into the reactivity of the interacting molecular fragments, based on their electronic density distribution.

The TTCA molecule has a specific symmetric structure, with three sulfur atoms bonded to a triazine ring. With respect to the tautomerism of TTCA, in the solid state the predominant form is that with three protonated nitro­gen atoms (Scheme 1) (Wzgarda-Raj *et al.*, 2021*a* ▸); this may be supported by the fact that the N—H group has relatively stronger hydrogen-bond-donor properties than the S—H counterpart (Gilli & Gilli, 2013 ▸).

A detailed analysis of the Cambridge Structural Database (CSD) by Wzgarda-Raj *et al.* (2021*b* ▸) found a cyclic 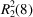 synthon (Bernstein *et al.*, 1995 ▸; Etter, 1990 ▸; Etter *et al.*, 1990 ▸) formed by N—H⋯S interactions to be the most characteristic for crystal structures with TTCA. This synthon is further propagated to linear or zigzag double chains, cyclic assemblies, and di-periodic structures. In this study, for all investigated multicomponent crystals, the neutral TTCA molecules or anions form centrosymmetric dimers that can be described as a mentioned above ring 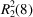 motif. As a result of N—H⋯N/N⋯H—N hydrogen bonds between TTCA dimers and imidazole moiety of drug molecules: MTZ, KTZ and MIC, the characteristic finite patterns can be found [Section 3.3; Figs. 2 ▸(*a*)–2 ▸(*c*)]. The common feature of the motifs observed in the crystal structures of salt, KTZ(+)·TTCA(−)·0.16H_2_O, and a cocrystal of salt, MIC·MIC(+)·TTCA(−), is a privileged site of N atom for proton transfer between the TTCA anion and the imidazole-based drug molecule with respect to the ring synthon.

**Figure 5:**
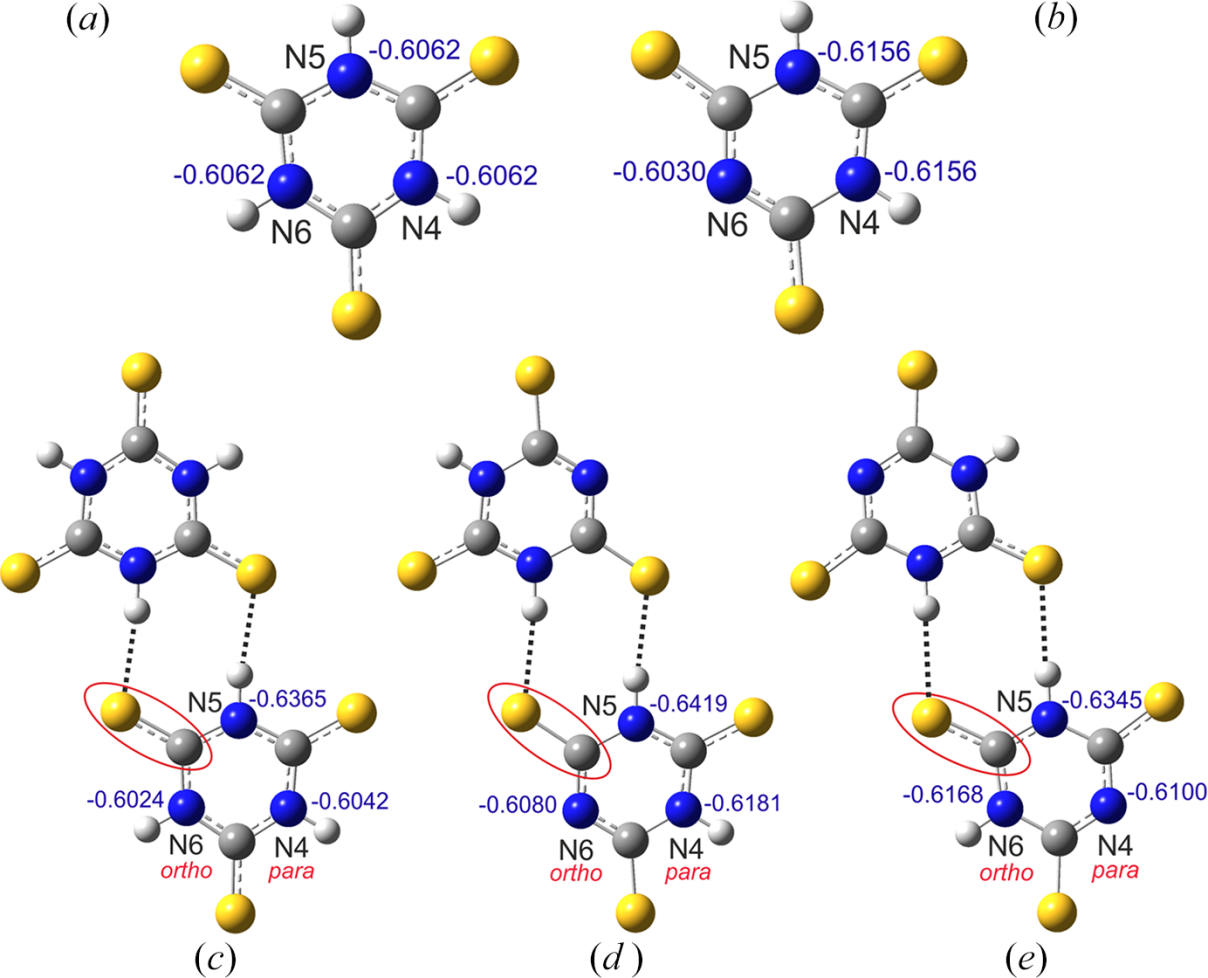
Optimized structures of (*a*) the tri­thio­cyanuric acid molecule, TTCA, and (*b*) an anion, TTCA(−), and as well as of the corresponding hydrogen-bonded dimers: (*c*) TTCA–TTCA, (*d*) TTCA(−)–TTCA(−)_*ortho* and (*e*) TTCA(−)–TTCA(−)_*para*. The natural atomic charges for nitro­gen atom are shown in blue.

The TTCA dimers built from neutral and ionized moieties were subjected to NBO analysis to gain a deeper understanding of their electronic structure. For a consistent comparison of the results, the analysis was performed on optimized structures of the neutral molecule and anion, and their corresponding dimers (Fig. 5 ▸). Two dimers formed from anions can be distinguished based on the site of deprotonated nitro­gen atom assigned as the *ortho* (N6 atom) or the *para* (N4 atom) positions with respect to the C—S bond involved in the hydrogen-bonded ring.

In all considered systems of TTCA, the nitro­gen atoms have the most electronegative character among all elements. The charge distribution analysis confirmed that the neutral molecule has high symmetry, and that each type of atom (S, N, C, H) have the same natural atomic charges (Table S1 in the supporting information). In comparison, TTCA(−) is a less symmetrical anion: one nitro­gen atom is deprotonated and has a higher atomic charge (−0.6030) *i.e.* less negative, than the other N atoms (−0.6156).

For two centrosymmetric dimers formed by anions, Figs. 5 ▸(*c*)–5 ▸(*e*), one can observe that the molecular symmetry of the anion is broken; among the three nitro­gen atoms, the maximum charge is found for the N5 atom, involved in the intermolecular hydrogen bond, and the minimum for the deprotonated nitro­gen atom. Applying the observed trends to the dimer formed from the neutral TTCA molecules, the discrimination of charges between N4 and N6 nitro­gen atoms can be used to predict the preferred site of proton transfer between TTCA and the base molecule; this site appears to be the *ortho* position in respect to C—S bond involved in the N—H⋯S hydrogen bond.

Interestingly, the M06L calculations indicate that the TTCA–TTCA(−)_*para* dimer is more stable than TTCA(−)–TTCA(−)_*ortho* by 0.15 kcal mol^−1^ in the gas phase, which is contrary to our observations in the crystalline state. It seems that the packing forces in crystals play a crucial role in the formation of centrosymmetric dimers from TTCA anions.

The preferred site for proton transfer between TTCA and other molecules can also be predicted by analysing the donor-acceptor orbital interactions within the NBO framework. This theory assumes that the interactions can be examined between the filled Lewis and empty non-Lewis orbitals. The strength of such interactions is quantified by the stabilization energy, *E*(2), whose highest value corresponds to the strongest interaction (Weinhold & Landis, 2001 ▸). It should be noted that the present study focuses only on the interactions involving the orbitals of the nitro­gen atoms. The *E*(2) values are presented in Table 6 ▸. For the neutral molecule of TTCA, the orbital interactions of three N atoms have the same stabilization energy values, and this is related to the molecular high symmetry, whereas the corresponding energy values *E*(2) slightly differ for the TTCA anion and all dimers (Table 6 ▸). As the effect of proton transfer can be showed by the smallest stabilization energy values (*i.e.* below 1 kcal mol^−1^), for the dimer TTCA–TTCA, it appears that proton transfer is most likely to occur at N6 atom, *i.e.* the atom involved in the weakest orbital interaction. However, as the energy difference Δ*E*(2) between σ(S1—C7)→σ*N6 and σ(S2−C8)**→**σ*N4 is not significant, it is possible that the transfer may also occur at N4 atom. It is highly unlikely for a proton transfer to occur at N5 atom, as its orbital is involved in the strongest interaction and N5 itself is characterized by the smallest atomic charge value [Fig. 5 ▸(*c*), Table S1].

## 4. Conclusion

The study describes the successful cocrystallization of imidazole-based drugs, namely metronidazole (MTZ), ketoconazole (KTZ), and miconazole (MIC) with tri­thio­cyanuric acid (TTCA), and presents the structural characterization of multicomponent crystals. Interestingly, three different forms were obtained: a cocrystal with MTZ, a salt with KTZ, and a hybrid, a cocrystal of salt with MIC. In the latter two cases, the proton being transferred from acid to base was localized in the difference Fourier map and was confirmed geometrically based on the analysis of the C—N—C angle of the imidazole ring.

For the three multicomponent adducts, the acid–base pair was formed by N—H⋯N hydrogen bonds between TTCA acid and an imidazole N atom; however, in the salt and cocrystal of salt, proton transfer resulted in the exchange of the roles of proton donor and proton acceptor. Regardless of whether proton transfer occurs or not, two acid–base pairs form a related secondary supramolecular motif consisting of TTCA molecules linked by a centrosymmetric N—H⋯S hydrogen bond.

Based on our analysis of supramolecular motifs combined with the analysis of atomic charges calculated for model TTCA systems, it can be seen that both acid–base pairs and the 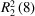 synthon for TTCA dimers coexist in a common finite pattern. The preferred position for the deprotonated N atom of TTCA is *ortho* with respect to the C—S bond involved in the N—H⋯S interaction; while the N atom in the *para* position retains its proton-donor character. In this way, all analysed crystals, regardless of their multicomponent form, are characterized by a related robust motif. This motif is further reproduced in the crystal network in various ways depending on the drug.

The robustness of supramolecular synthons is a critical issue in crystal engineering. As such, our study of the imidazole-based drugs and TTCA molecules provides greater insight into the complex intermolecular forces shaping the architecture of pharmaceutical cocrystals and salts.

## Supplementary Material

Crystal structure: contains datablock(s) global, MTZ-TTCA, KTZ-TTCA-0.16H2O, 2MIC-TTCA. DOI: 10.1107/S2052520624005055/aw5087sup1.cif

Structure factors: contains datablock(s) MTZ-TTCA. DOI: 10.1107/S2052520624005055/aw5087MTZ-TTCAsup2.hkl

Structure factors: contains datablock(s) KTZ-TTCA-0.16H2O. DOI: 10.1107/S2052520624005055/aw5087KTZ-TTCA-0.16H2Osup3.hkl

Structure factors: contains datablock(s) 2MIC-TTCA. DOI: 10.1107/S2052520624005055/aw50872MIC-TTCAsup4.hkl

Figs S1-S9, Tables S1-S6. DOI: 10.1107/S2052520624005055/aw5087sup5.pdf

CCDC references: 2358900, 2358901, 2358902

## Figures and Tables

**Table 1:** Experimental details

	MTZ–TTCA	KTZ–TTCA·0.16H_2_O	2MIC–TTCA
Crystal data			
Chemical formula	C_6_H_9_N_3_O_3_·C_3_H_3_N_3_S_3_	C_26_H_29_Cl_2_N_4_O_4_·C_3_H_2_N_3_S_3_·0.16H_2_O	C_18_H_14_Cl_4_N_2_O·C_18_H_15_Cl_4_N_2_O·C_3_H_2_N_3_S_3_
*M*_r_	348.43	711.57	1009.49
Crystal system, space group	Monoclinic, *P*2_1_/*c*	Monoclinic, *P*2_1_/*c*	Triclinic, *P* 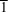
Temperature (K)	293	295	293
*a*, *b*, *c* (Å)	14.4914 (1), 5.1924 (1), 19.8863 (1)	13.0291 (1), 10.9297 (1), 23.8029 (3)	8.7764 (3), 15.8851 (6), 16.5229 (6)
α, β, γ (°)	90, 91.457 (1), 90	90, 102.897 (1), 90	88.869 (3), 80.569 (3), 74.929 (3)
*V* (Å^3^)	1495.86 (3)	3304.12 (6)	2193.60 (14)
*Z*	4	4	2
Radiation type	Cu *K*α	Cu *K*α	Cu *K*α
μ (mm^−1^)	4.73	3.93	6.40
Crystal size (mm)	0.22 × 0.11 × 0.06	0.17 × 0.14 × 0.03	0.25 × 0.04 × 0.03

Data collection			
Diffractometer	XtaLAB Synergy, Dualflex, HyPix	XtaLAB Synergy, Dualflex, HyPix	XtaLAB Synergy, Dualflex, HyPix
Absorption correction	Gaussian (*CrysAlis PRO*)	Gaussian (*CrysAlis PRO*)	Gaussian (*CrysAlis PRO*)
*T*_min_, *T*_max_	0.418, 1.000	0.240, 1.000	0.457, 1.000
No. of measured, independent and observed [*I* > 2σ(*I*)] reflections	23886, 3023, 2865	31878, 6692, 6113	24296, 8371, 6908
*R*_int_	0.025	0.033	0.033
(sin θ/λ)_max_ (Å^−1^)	0.633	0.633	0.617

Refinement			
*R*[*F*^2^ > 2σ(*F*^2^)], *wR*(*F*^2^), *S*	0.026, 0.073, 1.06	0.057, 0.131, 1.13	0.048, 0.142, 1.07
No. of reflections	3023	6692	8371
No. of parameters	207	487	544
No. of restraints	0	32	0
H-atom treatment	H atoms treated by a mixture of independent and constrained refinement	H atoms treated by a mixture of independent and constrained refinement	H atoms treated by a mixture of independent and constrained refinement
Δρ_max_, Δρ_min_ (e Å^−3^)	0.16, −0.20	0.44, −0.48	0.58, −0.45

Computer programs: *CrysAlis PRO* (Rigaku OD, 2023 ▸), *SHELXT* (Sheldrick, 2015*a* ▸), *SHELXL2019/2* (Sheldrick, 2015*b* ▸), *Mercury* (Macrae *et al.*, 2020 ▸), *PLATON* (Spek, 2020 ▸), *publCIF* (Westrip, 2010 ▸).

**Table 2:** Hydrogen-bond geometry (Å, °) for MTZ·TTCA

*D*—H⋯*A*	*D*—H	H⋯*A*	*D*⋯*A*	*D*—H⋯*A*
O1—H1⋯O3^i^	0.72 (2)	2.15 (2)	2.8462 (14)	162 (2)
N7—H7⋯O1^ii^	0.806 (18)	1.948 (19)	2.7447 (14)	169.9 (18)
N5—H5⋯N2	0.849 (19)	2.14 (2)	2.9891 (17)	173.5 (17)
N6—H6⋯S3^iii^	0.82 (2)	2.55 (2)	3.3618 (12)	171.3 (17)
C4—H4⋯S1	0.93	2.83	3.5291 (14)	133

Symmetry codes: (i) −*x* + 1, *y* − ½, −*z* + ½; (ii) *x*, −*y* − ½, *z* − ½; (iii) −*x*, −*y* − 2, −*z*.

**Table 3:** Hydrogen-bond geometry (Å, °) for KTZ(+)·TTCA(−)·0.16H_2_O

*D*—H⋯*A*	*D*—H	H⋯*A*	*D*⋯*A*	*D*—H⋯*A*
N2—H2⋯N5	0.92 (4)	1.82 (4)	2.742 (3)	175 (4)
N6—H6⋯S3^i^	0.87 (3)	2.46 (4)	3.320 (3)	172 (3)
N7—H7⋯O4^ii^	0.81 (3)	1.99 (3)	2.803 (4)	177 (3)
C8—H8⋯O5^iii^	0.93	2.33	3.18 (2)	152
C12*A*—H12*B*⋯O5	0.97	2.36	3.19 (3)	144
C12*B*—H12*C*⋯O5	0.97	1.98	2.73 (3)	133
C13*B*—H13*B*⋯O5	0.98	2.22	2.89 (3)	124
C21—H21*A*⋯O3*A*^iv^	0.97	2.59	3.507 (7)	158
O5—H5*A*⋯O4^iv^	0.87	1.96	2.75 (2)	151
O5—H5*B*⋯S1^v^	0.85	2.33	3.15 (2)	163

Symmetry codes: (i) −*x* + 1, −* y*, −*z*; (ii) −*x* + 2, −*y* + 1, −* z *; (iii) −*x*, *y* − ½, −*z* + ½; (iv) −*x* + 1, −*y* + 1, −*z*; (iv) −*x* + 1, −* y* + 1, −*z*; (v) *x* − 1, *y*, *z*.

**Table 4:** Hydrogen-bond geometry (Å, °) for MIC·MIC(+)·TTCA(−)

*D*—H⋯*A*	*D*—H	H⋯*A*	*D*⋯*A*	*D*—H⋯*A*
N4—H4*A*⋯N7	0.81 (4)	1.95 (4)	2.745 (3)	167 (4)
N5—H5*A*⋯N2	0.87 (3)	1.96 (3)	2.825 (3)	172 (3)
N6—H6*A*⋯S3^i^	0.83 (3)	2.55 (3)	3.369 (2)	172 (3)
C2—H2*A*⋯S1^ii^	0.97	2.81	3.772 (3)	173
C5—H5⋯Cl6^iii^	0.93	2.84	3.746 (3)	164
C22—H22*A*⋯S1^iv^	0.97	2.75	3.692 (3)	164

Symmetry codes: (i) −*x* + 1, −*y*, −*z* + 1; (ii) *x* + 1, *y*, *z*; (iii) −*x*, −*y* + 1, −*z* + 1; (iv) *x* − 1, *y*, *z*.

**Table 5:** Interaction energies (kJ mol^−1^) for selected molecular pairs *E*_tot_ is the total energy and its individual components: *E*_ele_ is electrostatic (*k* = 1.057), *E*_pol_ is polarization (*k* = 0.740), *E*_dis_ is dispersion (*k* = 0.871), *E*_rep_ is repulsion (*k* = 0.618).

Structure	Molecular pair	Interaction	*k* *E*_ele_	*k* *E*_pol_	*k* *E*_dis_	*k* *E*_rep_	*E*_tot_
MTZ·TTCA	TTCA–MTZ	N5—H5⋯N2	−64.4	−8.3	−15.2	40.1	−47.7
	TTCA–MTZ	N7—H7⋯O1^ii^	−67.3	−9.2	−17.2	44.4	−49.4
	TTCA–TTCA	N6—H6⋯S3^iii^	−55.7	−5.8	−13.1	48.1	−26.6
	MTZ–MTZ	O1—H1⋯O3^i^	−45.2	−7.1	−19.1	26.5	−45.0
KTZ(+)·TTCA(−)·0.16H_2_O	KTZ(+)–TTCA(−)	N2—H2⋯N5	−392.4	−60.3	−37.28	72.5	−417.6
	TTCA(−)–KTZ(+)	N7—H7⋯O4^ii^	−129.2	−8.8	−15.8	38.4	−115.4
	TTCA(−)–TTCA(−)	N6—H6⋯S3^i^	128.2	−18.1	−13.1	61.2	158.3
MIC·MIC(+)·TTCA(−)	TTCA(−)–MIC	N5—H5*A*⋯N2	−61.2	−19.2	−19.9	57.0	−43.4
	MIC(+)–TTCA(−)	N4—H4*A*⋯N7	−396.0	−55.2	−22.9	67.24	−407.0
	TTCA(−)–TTCA(−)	N6—H6⋯S3^i^	132.7	−16.8	−12.4	51.48	155.0

Symmetry codes: MTZ·TTCA (i) −*x* + 1, *y* − ½, −*z* + ½; (ii) *x*, −*y* − ½, *z* − ½; (iii) −*x*, −*y* − 2, −*z*; KTZ(+)·TTCA(−)·0.16H_2_O (i) −*x* + 1, −*y*, −*z*; (ii) −*x* + 2, −*y* + 1, −*z*; MIC·MIC(+)·TTCA(−) (i) −*x* + 1, −*y*, −*z* + 1.

**Table 6:** *E*(2) energies (kcal mol^−1^) obtained from the NBO calculations observed in the optimized molecules and dimers of TTCA σ is bonding natural orbital and σ* is antibonding natural orbital. 1 kcal mol^−1^ = 4.184  kJ mol^−1^.

		Stabilization energy *E*(2)				
Donor (*i*)	Acceptor (*j*)	TTCA	TTCA(−)	TTCA–TTCA	TTCA(−)–TTCA(−)_*ortho*	TTCA(−) –TTCA(−)_*para*
σ→σ* transitions						
σ(S1—C7)	σ*N6	0.89	0.92	0.62	0.88	1.34
σ(S2—C8)	σ*N4	0.89	1.24	1.15	1.16	0.99
σ(S3—C9)	σ*N5	0.89	1.40	1.55	1.00	1.39
