# Supplementary material for: Supramolecular architectures in multicomponent crystals of imidazole-based drugs and tri­thio­cyanuric acid
Source: Acta Crystallogr B Struct Sci Cryst Eng Mater. 2024 Jul 1;80(Pt 4):294–304. doi: 10.1107/S2052520624005055 (PMC11301895; doi:10.1107/S2052520624005055)
Supplement: Supplementary file 5 [file b-80-00294-sup5.pdf]

STRUCTURAL SCIENCE
CRYSTAL ENGINEERING
MATERIALS

Volume 80 (2024)

Supporting information for article:

Supramolecular architectures in multicomponent crystals of imidazole-based drugs and trithiocyanuric acid

Anna Ben, Marta Hoelm and Lilianna Chęcińska

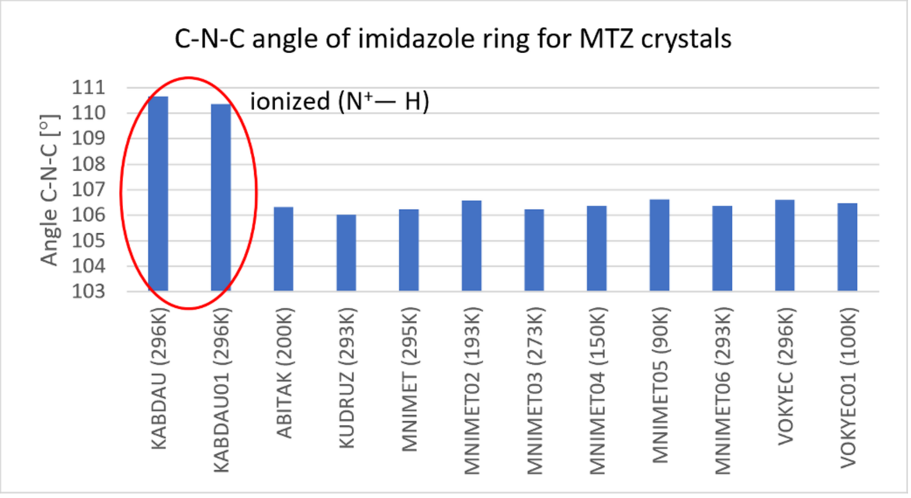

Figure S1 The C–N–C angle of imidazole ring for MTZ crystals (CSD ver. 5.44 September 2023).

[KABDAU]: Wang, Y., Chu, X., Yan, S. & Tang. G. (2010). *Acta Cryst*. E**66**, o2647.

[KABDAU01]: Rogers, R. (2020). CSD Communication (Private Communication).

[ABITAK]: Li, J., Hao, X., Wang, C., Liu, H., Liu, L., He, X. & Sun, C. C. (2021). *Pharmaceutics*, **13**, 546.

[KUDRUZ]: Zheng, K., Gao, S., Chen, M., Li, A., Wu, W., Qian, S. & Pang, Q. (2020). *CrystEngComm*, **22**, 1404.

[MNIMET]: Blaton, N. M., Peeters, O. M. & De Ranter C. J. (1979). *Acta Cryst.* B**35**, 2465.

[MNIMET02]: Galvan-Tejada, N., Bernes, S., Castillo-Blum, S. E., Noth, H., Vicente, R. & Barba-Behrens, N. (2002). *J. Inorg. Biochem*. **91**, 339.

[MNIMET03]: Yousuf, S. (2013). CSD Communication (Private Communication).

[MNIMET04]: Coles, S. J., Hursthouse, M. B., Maurya, M. R. & Azam, A. (2008). University of Southampton, *Crystal Structure Report Archive*, 606.

[MNIMET05]: Kalaiarasi, C., Christy, G., Gonnade, R. G., Hathwar, V. R. & Kumaradhas, P. (2019). *Acta Cryst.* B**75**, 942.

[MNIMET06]: Parsekar, N. U., Narvekar, K. U. & Srinivasan, B. R. (2020). CSD Communication (Private Communication).

[VOKYEC]: Zheng, K., Li, A., Wu, W., Qian, S., Liu, B., Pang, Q. (2019). *J. Mol. Struct*. **1197**, 727.

[VOKYEC01]: Seera, R. & Row, T. N. G. (2020). *Cryst. Growth Des*. **20**, 4667.

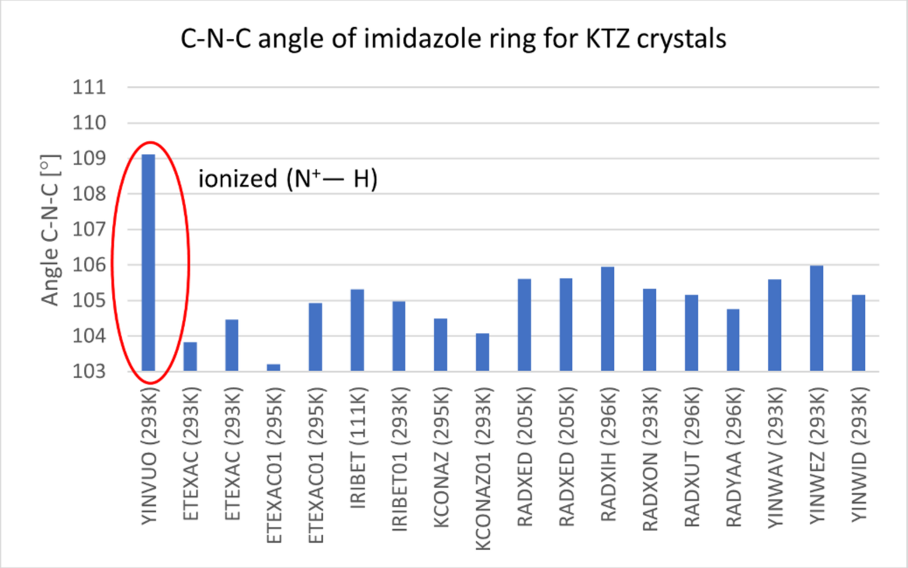

Figure S2 The C–N–C angle of imidazole ring for KTZ crystals (CSD ver. 5.44 September 2023).

[YINVUO]; [YINWAV]; [YINWEZ]; [YINWID]: Martin, F. A., Pop, M. M., Borodi, G., Filip, X. & Kacso, I. (2013). *Cryst. Growth Des*. **13**, 4295.

[ETEXAC]: Peeters, O. M., Blaton, N. M., Gerber, J. G. & Gal, J. (2004). *Acta Cryst.* E**60**, 367.

[ETEXAC01]: Perez-Benitez, A., Nieto, I. A. & Bernes, S. (2021). CSD Communication (Private Communication).

[IRIBET]; [IRIBET01]: Martin, F., Pop, M., Kacso, I., Grosu, I. G., Miclaus, M., Vodnar, D., Lung, I., Filip, G. A., Olteanu, E. D., Moldovan, R., Nagy, A., Filip, X. & Baldea, I. (2020). *Mol. Pharmaceutics*, **17**, 919.

[KCONAZ]: Peeters, O. M., Blaton, N. M. & De Ranter, C. J. (1979). *Acta Cryst.* B**35**, 2461.

[KCONAZ01]; [RADXED]; [RADXIH]; [RADXON]; [RADXUT]; [RADYAA]: Chen, X., Li, D., Deng, Z. & Zhang, H. (2020). *Cryst. Growth Des*. **20**, 6973.

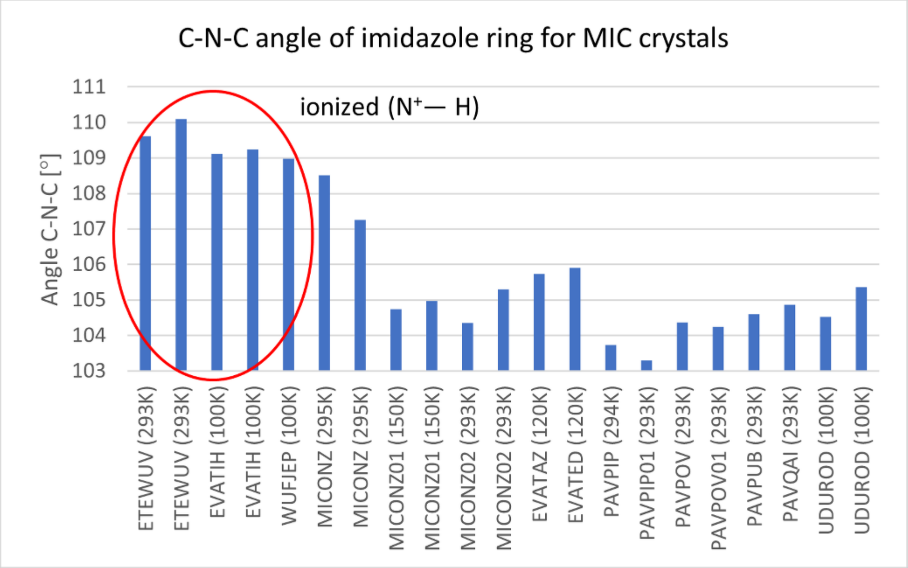

Figure S3 The C–N–C angle of imidazole ring for MIC crystals (CSD ver. 5.44 September 2023).

[ETEWUV]: Peeters, O. M., Blaton, N. M., Aeschlimann, C. & Gal, J. (2004). *Acta Cryst.* E**60**, 365.

[WUFJEP]: Patel, M. A., Luthra, S., Shamblin, S. L., Arora, K., Krzyzaniak, J. F. & Taylor, L. S. (2018). *Mol. Pharmaceutics*, **15**, 40.

[MICONZ]: Peeters, O. M., Blaton, N. M. & De Ranter, C. J. (1979). *Bull. Soc. Chim. Belg*. **88**, 265.

[MICONZ02]; [PAVPIP01]; [PAVPOV01]: Kaspiaruk, H. & Chęcińska, L. (2022). *Acta Cryst.* C**78**, 343.

[EVATIH]; [MICONZ01]; [EVATAZ]; [EVATED]: Drozd, K. V., Manin, A. N., Voronin, A. P., Boycov, D. E., Churakov, A. V. & Perlovich, G. L. (2021). *Phys. Chem. Chem. Phys.* **23**, 12456.

[PAVPIP]; [PAVPOV]; [PAVPUB]; [PAVQAI]: Panini, P., Boel, E., Van Meervelt, L. & Van den Mooter, G. (2022). *Cryst. Growth Des*. **22**, 2703.

[UDUROD]: Kersten, K. M., Breen, M. E., Mapp, A. K. & Matzger, A. J. (2018). *Chem. Commun*. **54**, 9286.

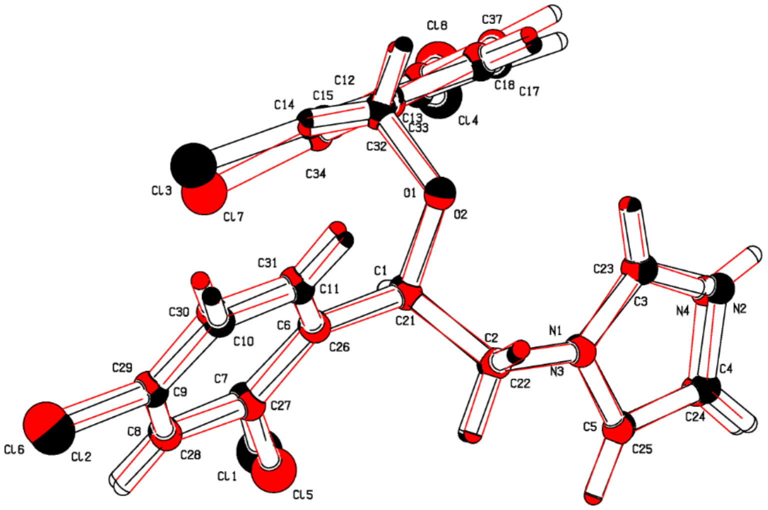

Figure S4 An overlay of two miconazole molecules from the asymmetric unit of MIC·MIC(+)·TTCA(-); the colour code is black = MIC and red = MIC(+).

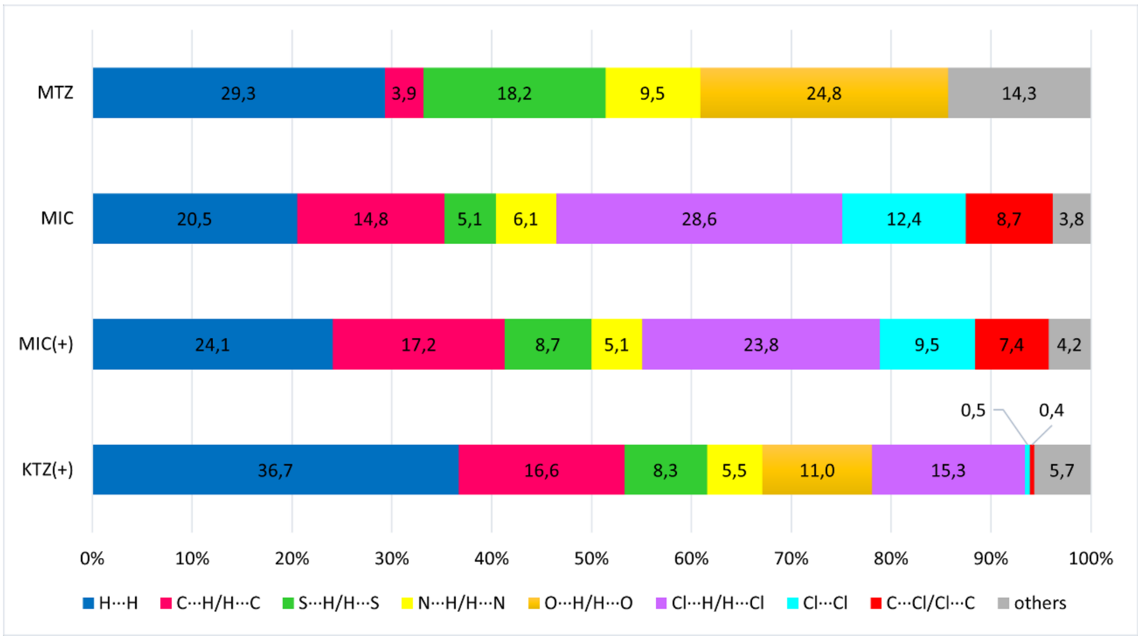

Figure S5 Diagram of the percentage contributions of various contacts to the Hirshfeld surface area of the imidazole-based drugs in the analysed crystals.

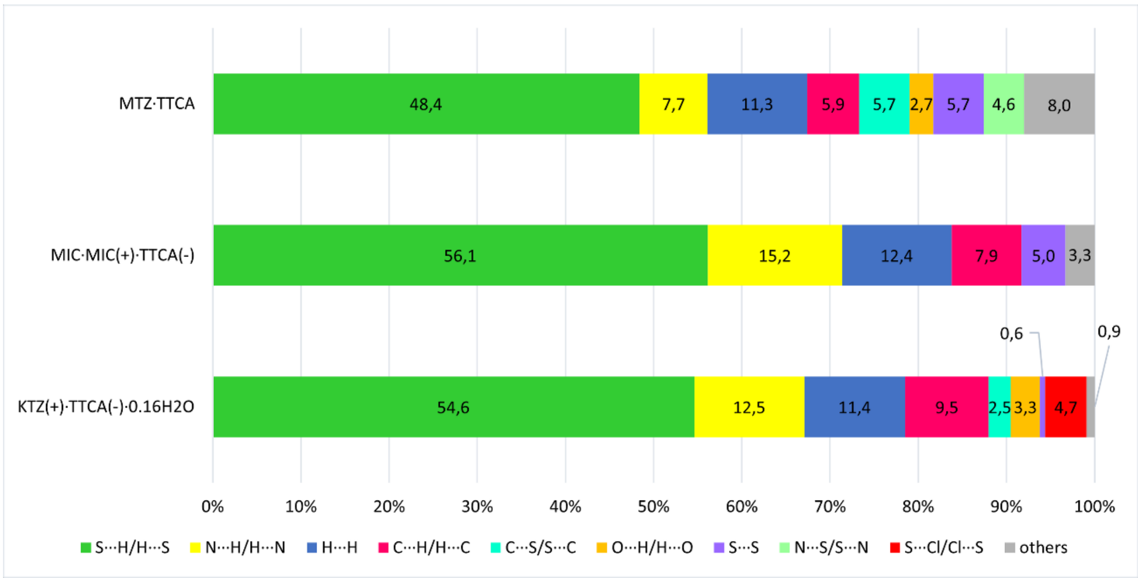

Figure S6 Diagram of the percentage contributions of various contacts to the Hirshfeld surface area of the trithiocyanuric acid in the analysed crystals.

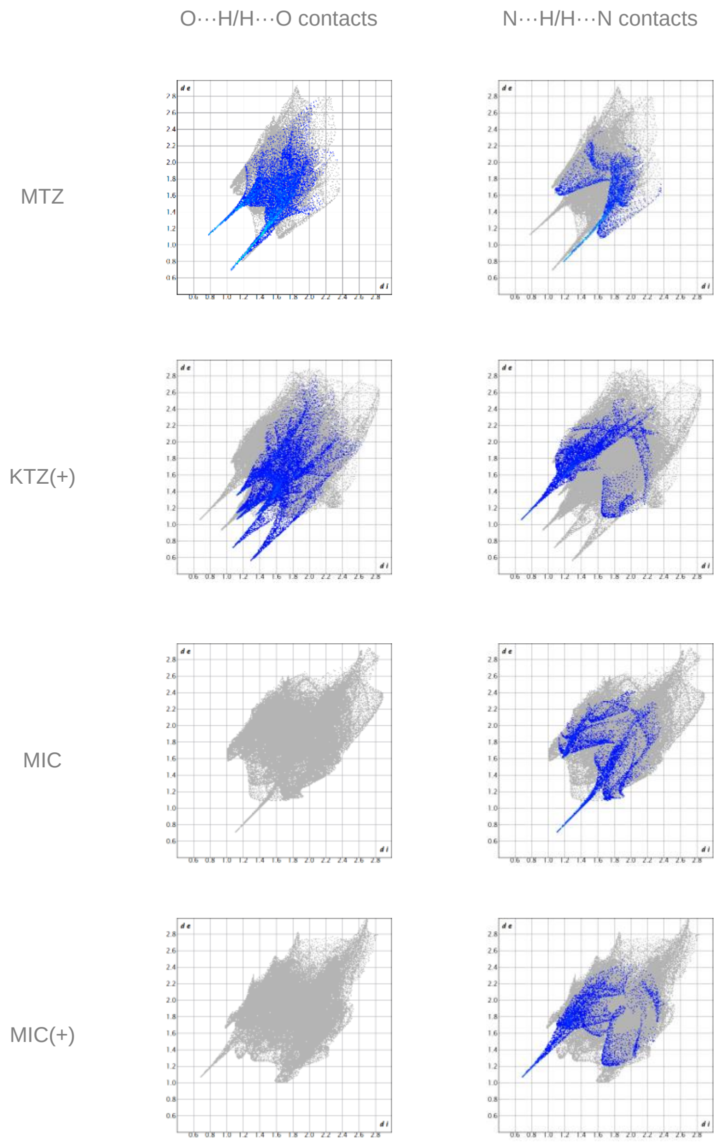

Figure S7 Fingerprint plots of O···H/H···O contacts and N···H/H···N contacts from the Hirshfeld surface to the nearest nucleus inside the surface (di) and outside the surface (de) calculated for the imidazole-based drug molecules in the analysed crystals.

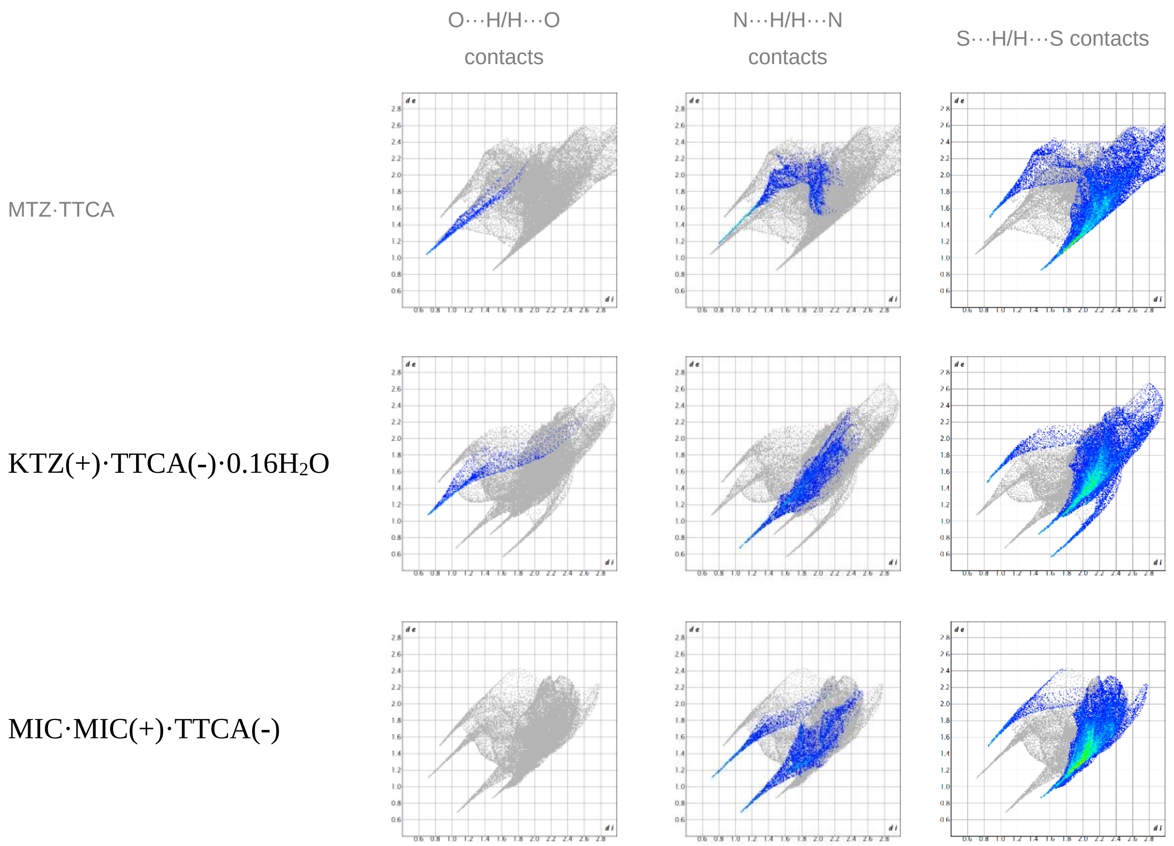

Figure S8 Fingerprint plots of O···H/H···O contacts, N···H/H···N contacts and S···H/H···S contacts from the Hirshfeld surface to the nearest nucleus inside the surface (di) and outside the surface (de) calculated for the trithiocyanuric acid in the analysed crystals.

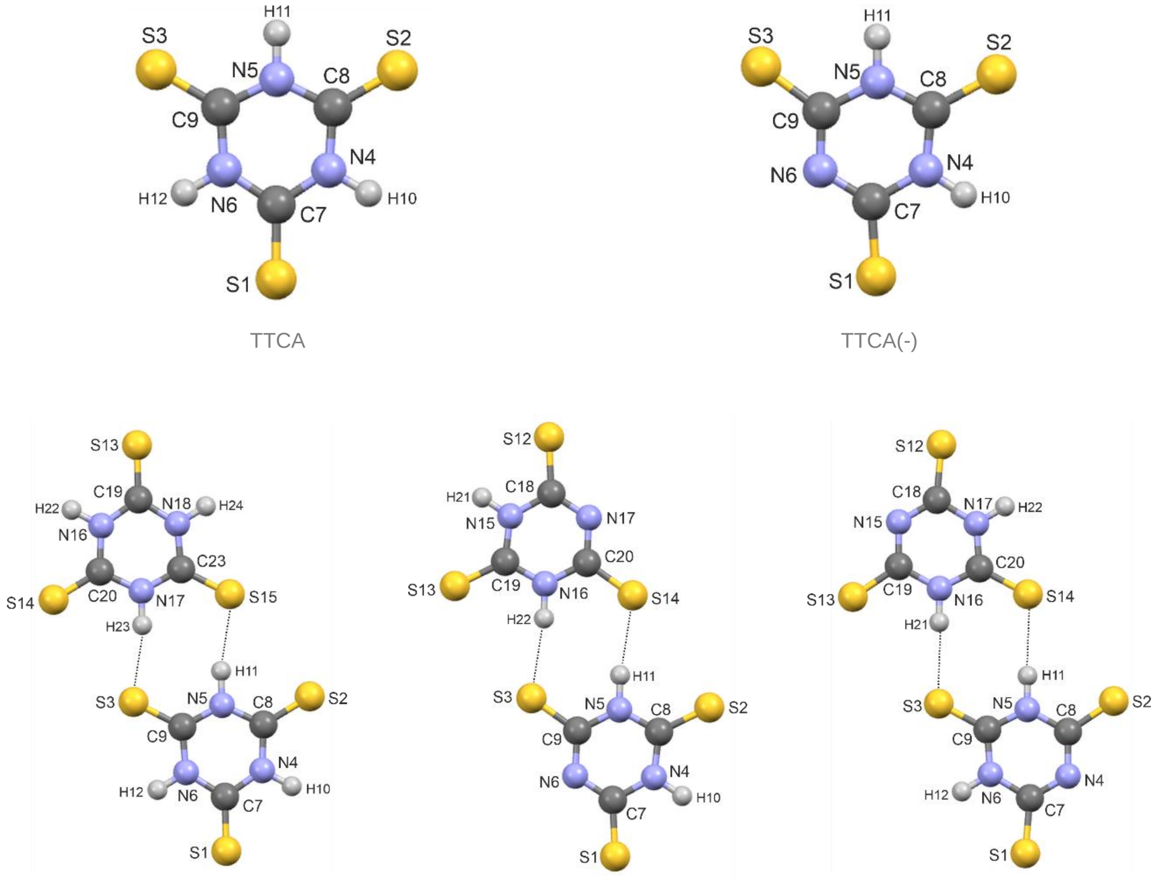

TTCA-TTCA TTCA(-)-TTCA(-)_*ortho* TTCA(-)-TTCA(-)_*para*

Figure S9 Optimized structures with atom-numbering schemes of the trithiocyanuric acid molecule, TTCA, and an anion, TTCA(-), and as well as of the corresponding hydrogen-bonded dimers: TTCA-TTCA, TTCA(-)-TTCA(-)_*ortho* and TTCA(-)-TTCA(-)_*para*.

Table S1 Natural atomic charges (NPA) calculated for optimized structures of TTCA.

All atoms	TTCA	TTCA(-)	TTCA-TTCA	TTCA(-)-TTCA(-) *_ortho*	TTCA(-)-TTCA(-) *_para*
S1	-0.07503	-0.27478	-0.07273	-0.33875	-0.33243
S2	-0.07503	-0.28121	-0.06371	-0.27172	-0.26605
S3	-0.07504	-0.27486	-0.10502	-0.23609	-0.24928
N4	-0.60617	-0.61557	-0.60416	-0.61810	-0.60995
N5	-0.60618	-0.61560	-0.63647	-0.64188	-0.63450
N6	-0.60620	-0.60304	-0.60235	-0.60798	-0.61677
C7	0.24906	0.26962	0.24929	0.28492	0.28289
C8	0.24904	0.30007	0.25050	0.30194	0.27226
C9	0.24905	0.26962	0.27893	0.27734	0.30198
H10	0.43217	0.41290	0.43158	0.40788	0.44168
H11	0.43215	0.41293	0.44213	0.44243	0.41019
H12	0.43218		0.43200		
S12/S13			-0.07273	-0.33875	-0.33243
S13/S14			-0.06371	-0.27172	-0.26605
S14/S15			-0.10502	-0.23609	-0.24928
N15/N16			-0.60416	-0.61810	-0.60995
N16/N17			-0.63647	-0.64188	-0.63450
N17/N18			-0.60235	-0.60798	-0.61677
C18/C19			0.24929	0.28492	0.28289
C19/C20			0.25050	0.30194	0.27226
C20/C21			0.27893	0.27734	0.30198
H21/H22			0.43158	0.40788	0.44168
H22/H23			0.44213	0.44243	0.41019
H24			0.43200		

Table S2 Final Cartesian coordinates (X, Y, Z in Å) for the gas-phase structure of TTCA optimized at the M06L/6-311++G(3df,3pd) level of theory.

Atom	X	Y	Z
S	-2.383086	-1.907983	0.000093
S	-0.461027	3.017726	-0.000059
S	2.844123	-1.109473	0.000230
N	-1.233080	0.481053	0.000361
N	1.033148	0.827204	-0.000120
N	0.199912	-1.308599	-0.000787
C	-1.109874	-0.888772	-0.000062
C	-0.214740	1.405505	0.000072
C	1.324633	-0.516978	-0.000150
H	-2.172412	0.847402	0.000592
H	1.820064	1.457481	-0.000043
H	0.352216	-2.305322	-0.000094

Table S3 Final Cartesian coordinates (X, Y, Z in Å) for the gas-phase structure of TTCA(-) anion optimized at the M06L/6-311++G(3df,3pd) level of theory.

Atom	X	Y	Z
S	2.673172	-1.495095	-0.000917
S	-0.000217	3.031120	-0.000384
S	-2.672952	-1.495470	0.000302
N	1.126103	0.615980	0.002794
N	-1.126194	0.615813	-0.001735
N	0.000107	-1.454788	0.000216
C	1.159131	-0.791059	0.000710
C	-0.000099	1.369242	0.000548
C	-1.159008	-0.791232	-0.000085
H	2.012035	1.095299	0.002716
H	-2.012357	1.095076	-0.002709

Table S4 Final Cartesian coordinates (X, Y, Z in Å) for the gas-phase structure of TTCA-TTCA dimer optimized at the M06L/6-311++G(3df,3pd) level of theory.

Atom	X	Y	Z
S	5.178069	3.468051	0.313108
S	0.546887	2.951285	2.809405
S	1.725201	-0.167391	-1.314650
N	2.808207	3.060114	1.435563
N	1.295053	1.477542	0.734735
N	3.306516	1.709325	-0.344318
C	3.732140	2.731986	0.474314
C	1.572575	2.483370	1.631010
C	2.109556	1.043595	-0.268789
H	3.060385	3.803518	2.068534
H	0.385706	1.014337	0.837287
H	3.942322	1.421926	-1.072245
S	-5.178069	-3.468051	-0.313108
S	-0.546887	-2.951285	-2.809405
S	-1.725201	0.167391	1.314650
N	-2.808207	-3.060114	-1.435563
N	-1.295053	-1.477542	-0.734735
N	-3.306516	-1.709325	0.344318
C	-3.732140	-2.731986	-0.474314
C	-1.572575	-2.483370	-1.631010
C	-2.109556	-1.043595	0.268789
H	-3.060385	-3.803518	-2.068534
H	-0.385706	-1.014337	-0.837287
H	-3.942322	-1.421926	1.072245

Table S5 Final Cartesian coordinates (X, Y, Z in Å) for the gas-phase structure of TTCA(-)-TTCA(-)_ortho dimer optimized at the M06L/6-311++G(3df,3pd) level of theory.

Atom	X	Y	Z
S	5.228251	3.526353	0.403596
S	0.603265	3.045000	2.823026
S	1.735180	-0.142096	-1.254273
N	2.859376	3.077415	1.432660
N	1.354663	1.529628	0.748727
N	3.428271	1.704337	-0.380406
C	3.770040	2.690326	0.445283
C	1.630432	2.524342	1.624960
C	2.232799	1.105093	-0.254691
H	3.130361	3.824748	2.050693
H	0.446323	1.057233	0.826135
S	-5.228251	-3.526353	-0.403596
S	-0.603265	-3.045000	-2.823026
S	-1.735180	0.142096	1.254273
N	-2.859376	-3.077415	-1.432660
N	-1.354663	-1.529628	-0.748727
N	-3.428271	-1.704337	0.380406
C	-3.770040	-2.690326	-0.445283
C	-1.630432	-2.524342	-1.624960
C	-2.232799	-1.105093	0.254691
H	-3.130361	-3.824748	-2.050693
H	-0.446323	-1.057233	-0.826135

Table S6 Final Cartesian coordinates (X, Y, Z in Å) for the gas-phase structure of TTCA(-)-TTCA(-)_para dimer optimized at the M06L/6-311++G(3df,3pd) level of theory.

Atom	X	Y	Z
S	5.291277	3.467101	0.233378
S	0.661619	3.066900	2.861442
S	1.738280	-0.091905	-1.242609
N	2.953820	3.223316	1.514974
N	1.385709	1.600055	0.805460
N	3.353197	1.782802	-0.295135
C	3.771396	2.821976	0.544283
C	1.755989	2.637709	1.676591
C	2.160176	1.137041	-0.201697
H	0.473858	1.161230	0.935907
H	3.983091	1.490390	-1.024595
S	-5.291277	-3.467101	-0.233378
S	-0.661619	-3.066900	-2.861442
S	-1.738280	0.091905	1.242609
N	-2.953820	-3.223316	-1.514974
N	-1.385709	-1.600055	-0.805460
N	-3.353197	-1.782802	0.295135
C	-3.771396	-2.821976	-0.544283
C	-1.755989	-2.637709	-1.676591
C	-2.160176	-1.137041	0.201697
H	-0.473858	-1.161230	-0.935907
H	-3.983091	-1.490390	1.024595
